# Understanding the Acceptance of an eHealth Technology in the Early Stages of Development: An End-User Walkthrough Approach and Two Case Studies

**DOI:** 10.2196/10474

**Published:** 2018-06-15

**Authors:** Lex van Velsen, Mirka Evers, Cristian-Dan Bara, Harm Op den Akker, Simone Boerema, Hermie Hermens

**Affiliations:** ^1^ Telemedicine Cluster Roessingh Research and Development Enschede Netherlands; ^2^ Biomedical Signals and Systems Group University of Twente Enschede Netherlands

**Keywords:** eHealth, acceptance, design, walkthrough, agile design

## Abstract

**Background:**

Studies that focus on the acceptance of an electronic health (eHealth) technology generally make use of surveys. However, results of such studies hold little value for a redesign, as they focus only on quantifying end-user appreciation of general factors (eg, perceived usefulness).

**Objective:**

We present a method for understanding end-user acceptance of an eHealth technology, early in the development process: The eHealth End-User Walkthrough.

**Methods:**

During a walkthrough, a participant is guided by using the technology via a scenario, a persona, and a low-fidelity protoype. A participant is questioned about factors that may affect acceptance during and after the demonstration. We show the value of the method via two case studies.

**Results:**

During the case studies, participants commented on whether they intend to use a technology and why they would (not) use its main features. They also provided redesign advice or input for additional functions. Finally, the sessions provide guidance for the generation of business models and implementation plans.

**Conclusions:**

The eHealth End-User Walkthrough can aid design teams in understanding the acceptance of their eHealth application in a very early stage of the design process. Consequently, it can prevent a mismatch between technology and end-users’ needs, wishes and context.

## Introduction

### Background

Every new technology that is being developed or introduced faces challenges concerning end-user acceptance. When the train was introduced in the early 19th century, people were reluctant to use it, as they were afraid their bodies would melt going that fast (30 km/hour). Also, when the telephone first became available, people were not eager to install one in their home, as they feared it would attract lightning. Although these examples are historical and seem funny now, we have to deal with similar issues today.

Having a clear overview of the facilitators and barriers towards use is crucial for technology design and the development of a successful implementation strategy. Electronic health (eHealth; “health services and information delivered or enhanced through the internet and related technologies” [1]) is no exception. Numerous studies have identified factors that determine end-user acceptance of specific eHealth technologies and applications. Perceived usefulness was found to affect physicians’ intention to use telemedicine [2]. Organizational facilitators were identified as the most important antecedent of healthcare professionals’ intention to use a telemonitoring application for chronic patients in primary care [3]. Perceived usefulness and self-efficacy came out as the two main drivers for Singaporean women’s intention to use smartphones for seeking health information [4]. Moreover, the acceptance of eHealth among patients with chronic respiratory diseases depends on disease specifics, demographics, and Information Communication Technology (ICT) use [5]. This list is, of course, only a snapshot of the available studies on the topic.

The majority of studies that focus on explaining the end-user acceptance of eHealth use the Technology Acceptance Model (TAM) [6] or the Unified Theory of Acceptance and Use of Technology (UTAUT) [7] as a basis [8,9]. Throughout the last decades, hundreds of variants of TAM and UTAUT were investigated for explaining technology acceptance, with a wide variety of adaptations in healthcare as well [10-13]. Surveys with rating scales are now the preferred data collection method. Despite their widespread use, the use of TAM and UTAUT-based surveys has also received critique. Several authors [14-16] argue that the results of these studies hold little value for developing implementation plans. The models focus predominantly on technological factors and not on the person or organizational characteristics. Next, the very general factors people use to explain the intention to use, such as perceived usefulness and perceived ease of use in TAM, are of little use to a technology design team [17-19]. They are very well suited to make a general overview of the beliefs and attitudes that affect the intention to use, but it is difficult to derive actionable (re)design advice from the findings of such studies. After all, a statement that a technology should be “useful” holds little value for system (re)design. What makes a technology useful? Should it allow a significant degree of control, or should it provide a specific feature? Without more actionable insights, results from these studies hold little practical value for technology design.

In this article, we present a method that can explore a wide range of previously unknown factors that may affect end-user acceptance of an eHealth technology in the early stages of the development process: the eHealth End-user Walkthrough (EEW). Applying the method, we posit, results in actionable results, as it can deliver redesign advice targeted at specific features or steps in the service model. TAM and UTAUT-based surveys, which are quantitative, confirmative methods, are unable to do so, as they can only state generally whether an eHealth technology is, for example, useful or easy to use. Using two case studies, we demonstrate its use and will answer our research question: how well does the EEW identify issues that hinder or facilitate end-user acceptance of a future eHealth technology?

This article is organized as follows. In the Methods section, we present a guide towards applying the method and discuss its place in the agile, human-centered design process for eHealth. The Results section includes the results of two case studies in which the method was applied. One study centered around the development of a large screen that acts as a central hub for disclosing eHealth applications for patients with a chronic disease or age-related impairments. The other one focused on the development of an online platform to support elderly knowledge workers in maintaining a healthy working routine. In the Discussion section, we reflect on the usefulness of the method in the two cases and discuss its advantages and drawbacks.

## Methods

### The eHealth End-User Walkthrough

**Figure 1:**
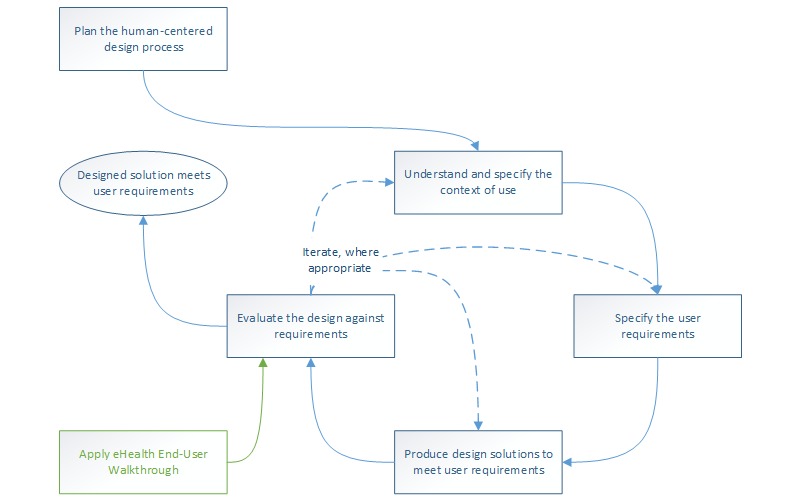
The human-centered design process and the place of the eHealth End-User Walkthrough (compare to ISO 9241-210 [21]).

The EEW is a method that allows a design team to quickly assess end-user acceptance of an eHealth application in the early phases of the design process. As such, it fits perfectly in a human-centered or agile design process. The latter has become very popular in recent years and advocates the continuous use of quick design-evaluation-redesign cycles [20]. Figure 1 shows the place of the EEW within the human-centered design process (as proposed by ISO standard 9241-210: Human-centered design for interactive systems [21]), namely as a way to evaluate the design regarding end-user acceptance.

During an EEW, a participant is presented with a simple prototype of the future technology, explained how the technology works, and questioned about relevant acceptance factors. To facilitate such an evaluation, the EEW builds forth on various design and evaluation methods (listed in Table 1).

During an EEW, the methods above are combined. A designated end-user of an eHealth technology is guided through its use employing a storyboard that shows how a persona uses the technology within the designated context of use. A participant is interviewed about factors that may inhibit or encourage acceptance, both during and after the demonstration. By combining these methods, the main functionalities of a future technology can be easily and vividly explained to novice end-users, while the data gathering methods are geared towards eliciting end-users’ opinion on these features.

### Conducting an eHealth End-User Walkthrough

**Figure 2:**
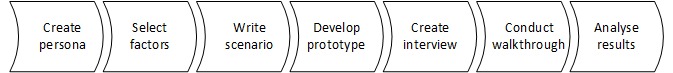
The main stages of preparing and conducting an eHealth End-User Walkthrough.

The following steps need to be taken to prepare an EEW (summarized in Figure 2).

First, one creates a persona that represents the designated end-user, or multiple personas when there are different types of primary end-users (eg, patients and care professionals, or older adults with and without cognitive impairments). For a practical guide on persona development for eHealth, see [27]. Second, the design team should select the most important factors that potentially affect end-user acceptance of the eHealth service. These can be derived from the standard models that explain technology acceptance (factors like ease of use and perceived usefulness), but we advise to focus on factors that are directly linked towards specific features or scenarios of use (eg, trust, controllability). Third, a scenario is written that describes the main features of the technology and the features that may profoundly influence the decision of the end-user to (not) accept a technology (eg, a feature that may be considered privacy-infringing). The scenario features the persona(s) from the first step.

**Table 1:** Methods that form the basis for an eHealth End-User Walkthrough.

Method	Description
Scenario development	A scenario is “a concrete description of an activity that the users engage in when performing specific tasks [22]” by use of the technology and takes a narrative form.
Personas	Personas are descriptions of fictitious users whose characteristics resemble the average for an end-user (sub)population [23]. They are often short and quite frivolous and used by the design team to talk about their end-users (reasoning that it’s easier to discuss what “Miriam” would like, rather than what “the average user” would like).
Storyboarding	A storyboard is a short, often graphical, narrative [24] which is cut into scenes (it is closely related to film-making).
Walkthroughs	During a walkthrough, potential end-users are presented prototypical screens of the final digital service and are asked to comment on them, mostly on their graphical design and usability [25].
Interviewing	Where discussions take place to elicit end-users’ rationale regarding the acceptance of specific technology features [26].

Fourth, a storyboard or low-fidelity prototype (either digital or on paper) is made. This strengthens the high-level scenario by providing visual representations of the features under investigation. Simple drawings or prototypes can provide suitable means to elicit feedback from end-users about the role each feature plays in the coming about of acceptance, without diverting to irrelevant details (such as the navigation within an eHealth application, which should be evaluated during a usability test with a high-fidelity prototype) [28,29]. Fifth and finally, an interview setup that questions acceptance factors is written. Factors that are linked to a specific feature or aspect of the scenario of use should be questioned whenever the feature or scenario-aspect comes up during the walkthrough. General factors (eg, perceived usefulness, willingness to pay) should be questioned at the end of the walkthrough.

Conducting an EEW can be done in a lab or at the home of a participant by a single researcher. Audio-recordings of the session will suffice for data analysis. During a session, the researcher should introduce the persona and should explain the technology by narrating the scenario. As soon as a main feature has been discussed, the researcher should pose the questions that relate to this feature. This way, all the main features of the technology should be introduced and discussed. At the end of a session, the researcher should question the participant about the general acceptance factors that the design team identified (step 2).

It is difficult, if not impossible, to state beforehand how many participants should be included in an EEW. This number depends on a range of factors, such as the diversity of the anticipated end-user population, the complexity of the technology under investigation, or the number of participants to which the design team has access. For the case of usability testing, it has been advised to include as many participants as budget and time allows while including 10 participants will ensure that the majority of critical issues will be identified [30]. We hypothesize that the same advice holds for an EEW.

The qualitative data collected should be analyzed systematically, in order to be of value for the design team. We recommend to transcribe the audio recordings and to apply inductive thematic analysis, whereby themes emerge from the data [31]. Analysis should have three goals. One, it should make clear how participants appreciate the main features that are presented during the EEW. Two, it should list the factors that affect this appreciation (what is also called functional analysis [32]). Three, participants’ intention to use the technology as a whole, and the factors that inhibit or driver this acceptance, should be uncovered (also called sensitizing concept analysis [32]). Ultimately, results should acknowledge the validity of the technology’s functional requirements or should allow to improve them.

### Case Study 1

**Figure 3:**
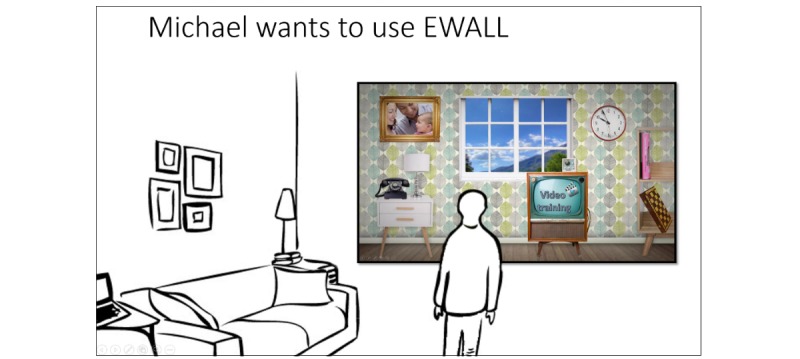
Early design sketch of the eWALL situated in a person’s home.

Funded under the European Commission’s FP7 framework, eWALL is a large-scale integration project in which various eHealth applications are delivered to older adults or patients with a chronic disease. The project’s primary objective was to create a “digital wall” that would allow end-users to access various health services easily. Focus was given to unobtrusively collecting health information from the patient as well as to provide feedback and coaching in those areas in which the user needed support the most. Using a large, touch-screen based user interface that was designed to look like a retro design living room, patients with a chronic disease or older adults with age-related impairments were able to self-manage their health [33]. The eWALL platform integrates different eHealth services, such as domotics, physical activity sensors, online web services, and medical devices (blood pressure and oxygen saturation measurement devices). They are then disclosed to the end-user via a single interface and single sign-on. Figure 3 displays how the design team envisioned eWALL to be present in the home of an end-user.

**Figure 4:**
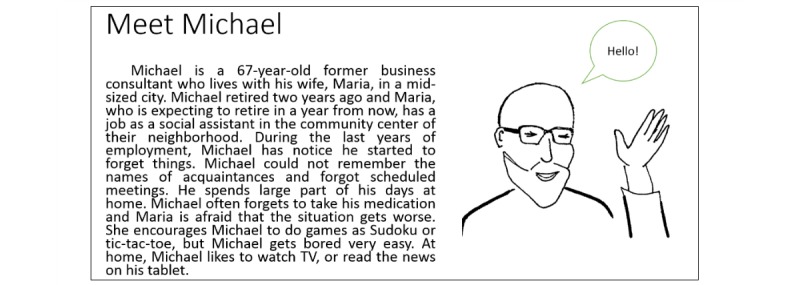
The eWALL persona Michael.

The end-user walkthrough of eWALL was supported by the persona of Michael (Figure 4). During a walkthrough, we questioned the participants, for each feature, about (1) their first impression of the feature, (2) whether the mock-up provided enough information to understand the functionality of the feature, and (3) their opinion of the feature in terms of relevant acceptance factors (eg, privacy, obtrusiveness of the technology).

At the end of the walkthrough, we questioned the participants about several acceptance factors concerning the total eWALL technology, like learnability and controllability. We also asked them to advise the developers.

**Figure 5:**
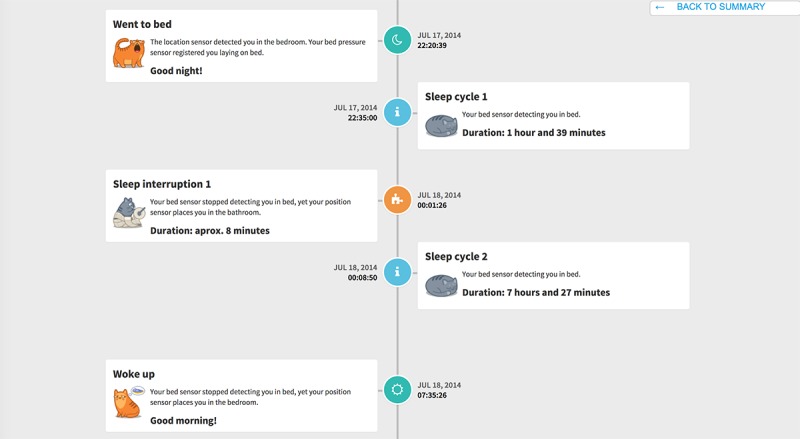
The eWALL sleep diary, whereby each episode of sleep or interruption of sleep is presented in text and cat icons.

As examples, we focus this case description on the participants’ appreciation of the eWALL main screen and sleep monitoring feature. The main screen displayed all of the eWALL functionalities, grouped into metaphors. Applications which mainly provide data overviews and monitoring outcomes are represented as books, while those which involve user, or smart home actions are represented as household items. It was shown to the participants, accompanied by the following explanation: “This is what Michael can see on his screen. He can touch the different items and open applications this way.” Then, we asked them “what do you think is behind each item on the screen?” After discussing the main screen, we took the participants along the different features of eWALL. When we embarked upon the sleep monitoring feature (which was depicted as a book, see Figure 5 for two pages within the book), we asked the participants a series of questions. The team of evaluators devised these questions to assess the general impression of participants (questions 1 and 2), to elicit factors that could affect acceptance of the feature (questions 3 and 4), and to question the topic of privacy (questions 5 and 6), which we anticipated would be important when deciding to accept the technology or not:

1. What is your first impression?
2. Do you think you understand what you are looking at?
3. Do you like what you see?
4. Do you think this information is useful?
5. How would it make you feel if this kind of information about you is collected?
6. Can you imagine that you would share this information with your family, your general practitioner, community nurse, or home care assistant?

Participants were recruited via a panel, consisting of older adults who indicated that they want to participate in research on the topic of technology for health.

### Case Study 2

Funded within the context of the European Active and Assisted Living (AAL) program, the Pearl project aimed to develop a suite of technologies to support older knowledge workers (aged 50 years and older). It sought to make them less sedentary, to help them to adopt a healthy physical activity pattern during working hours and to remain cognitively fit. This was done through task management functionalities, cognitive games, a physical exercise prompter, etc. All features could be accessed via a PC desktop application or a smartphone app. Data was collected by means of activity sensors, mobile phone prompts, and the digital agenda of the end-user.

During an EEW, participants were guided through the technology via the story of the persona Suzy. Then, with each participant per Pearl feature we discussed the following topics (1) their first impression of the feature, (2) whether the mock-up provided enough information to understand the functionality of the feature, and (3) whether the feature meets the expectations of the participants regarding its functionality. We posed questions (1) and (2) to assess the first, general impression of the feature. With question (3) we aimed to elicit factors that might hinder or facilitate acceptance of the specific feature. After walking through all the different features, we questioned the participants about the Pearl system in general, including aspects like their intention to use the technology and the preferred mode of introduction (prescribed by the employer, only upon the employee’s request). This way, we aimed to elicit factors that affect end-user acceptance of the Pearl technology as a whole.

**Figure 6:**
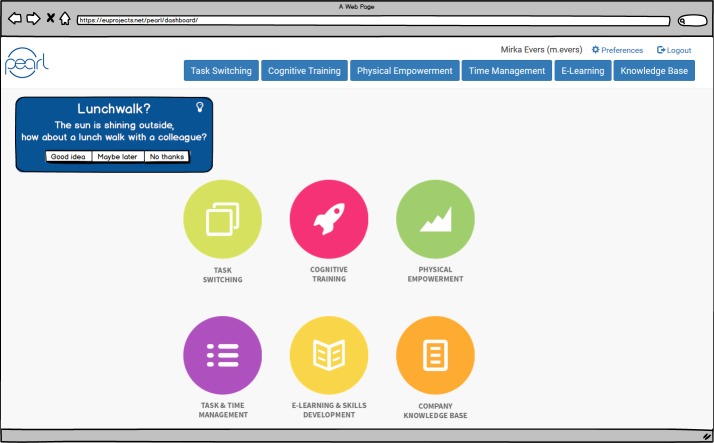
Mock-up of Pearl’s main interface, including a physical exercise suggestion

As an example, we discuss the participants’ appreciation of Pearl’s exercise prompter. This feature provides an end-user with suggestions for physical exercise after a period of physical inactivity at the workplace and when the digital agenda of the end-user indicates that there is no ongoing appointment or activity (Figure 6). At this point, we told the participants that “Because Suzy worked very hard all morning, she forgot to be physically active now and then. The Pearl system has noticed this long period of sitting, and that is why it suggests for a physical exercise. These suggestions are only given because Suzy indicated in her system settings that she thinks it is important to remain physically fit.” Participants were recruited via a convenience sample and consisted of co-workers of 50 years and older. They were not involved in the Pearl project, nor had they any knowledge of it beforehand.

### Ethics

The cases studies that are reported on in this article were exempt from Medical Ethical Approval by the Medical Ethical Committee Twente, the Netherlands. All participants provided informed consent, before their participation.

## Results

### Case Study 1

A total of 8 persons with age-related impairments, living in the surroundings of Enschede, the Netherlands, took part in the end-user walkthrough of eWALL. From these 5/8 (63%) of them were male, 3/8 (38%) were female. Their ages ranged from 66 to 88 years. In this results section, we report the participants’ reactions towards the main screen and sleep monitoring feature of eWall (as examples of the complete walkthroughs). Finally, we provide participants’ answers to the closing questions of each session.

#### Reactions Towards eWall’s Main Screen

All participants associated the different items on the main screen with the correct functionality, except for the window (which displays the weather conditions outside the end-user’s home). The participants did not associate the weather in the window with the actual weather outside. The participants also gave other suggestions (1) to show family members (especially grandchildren) in the picture frame, (2) to remove the phone (as it was too old-fashioned), and (3) to show upcoming appointments, reminders for taking medications, and mealtimes in the clock.

#### Appreciation of eWall’s Sleep Monitoring Feature

When we questioned the participants about their first, general impression, most of them replied that they thought positively of the sleep monitoring feature (it is noteworthy that none of them had chronic sleep problems). Privacy (the possibility to control who is and who is not allowed to view personal information) arose immediately as a primary concern. Two participants did not feel comfortable with the idea of being monitored while sleeping. The remaining six participants were fine with being observed but would restrict sharing this information to close family and their general practitioner.

I don't mind (if this information is collected). But when it's saved and it is useful for a doctor or someone similar, I would like to share this.

Male participant, 67 years

Then, we questioned the participants’ intention to use this feature. We did this to elicit factors that could affect acceptance. The majority of the participants indicated that they would not use the sleep monitoring functionality on a daily basis. They stated that they know themselves whether or not they slept well, and do not need technology for this. Finally, the participants suggested several improvements for the interface, such as summarizing nightly awakenings and sleep periods (instead of displaying them one by one) and making it possible to view last night’s information in one screen (thereby preventing the need to scroll).

#### Participants’ Replies to Closing Questions

At the end of the walkthrough, we discussed the use of eWall in general. First, we asked participants about the anticipated ease of use of using eWall in general (their thoughts on how difficult they expected it to be when (learning to) work with eWall). All participants indicated that they thought learning to work with eWALL would be easy. Next, we asked them whether they thought the technology would be easy to use in practice. Reactions were mixed. Most persons stated they thought it would be easy, the others did not think so. They attributed this to a lack of motivation to log their data and to use the technology diligently.

(Whether or not you use eWall) depends on how consequent a person is. You need to be motivated and disciplined.

Male participant, 67 years

As we were specifically interested in the issue of controllability (the extent to which an end-user can determine him or herself what the technology does), we asked the participants explicitly about their thoughts on this topic. Most participants were convinced they would be able to control eWALL, while some participants were unsure. When we questioned the intention to use the technology as a whole, some participants indicated that they would like to use eWALL. For example, one person indicated he would do so when he is homebound, while some participants were indecisive, and others stated they would not like to use eWALL (because they thought it was dull and should be improved, or because the visuals were not appealing). The participants had different advice for the developers. These included the ability to personalize the contents of the picture frame (eg, to display familiar pictures or persons, for people with cognitive decline). Some participants asked for a different visual style (stating that the current one was old-fashioned). There was one participant that sought the guarantee that the data would be stored safely (not accessible to outsiders). Other participants gave advice for successful implementation (only offer it in situations where somebody needs support, and to provide proper training before installation).

### Case Study 2

In total, 6 older office workers, working as knowledge workers in the surroundings of Enschede, the Netherlands, participated in the end-user walkthrough of the Pearl technology. Of these 5/6 (83%) were male, 1/6 (17%) was female, and collectively they had a mean age of 53 years (SD 10.8 years). In this section, we present Pearl’s exercise prompter functionality and the closing questions of each session.

#### Participants’ Appreciation of the Pearl Exercise Prompter

First, we questioned the participants’ first impression of the feature. Most participants had a good first impression of the exercise prompter. When asked about the understandability of the feature, the participants stated that the activity suggestion, generated by the prompter, was clearly illustrated and formulated. They particularly valued the option to unobtrusively receive these physical activity suggestions via their mobile phone (ie, not disturbing their ongoing work). Also, the three answering options: “Good idea,” “Maybe later,” and “No thanks,” were perceived quite well because it left them the choice of whether they wanted to adhere to the suggestion or not. Finally, we asked the participants whether the feature meets their expectations. Most participants expected that the prompter can help them to be more aware of their current physical activity behavior and consequently, become more active during the working day.

You get a short notification that you need to take a break. The sun is shining, so go for a walk. Maybe you won’t pay any attention to it. But I know I actually need to do this. Maybe when I get this in front of me on my screen, that I think: yes, I have to do this. […] Because I know it all, but I don’t do it. I’ll just do this, and I’ll just do that. And before you know it, an hour has passed already and you won’t do it anymore.

Female participant

The participants also gave several recommendations for improving the functionality, like indicating the time it takes to complete the suggested physical exercise, making it possible for the end-user to indicate whether s/he wants to exercise alone or with colleagues, adding functionality that makes it easier to organize a lunch walk, and suggestions should be explicitly linked towards physical activity goals (eg, steps to be made on a day).

#### Participants’ Replies to Closing Questions

participants indicated that they would like to use the Pearl system once available. Those that were willing to do so, especially liked the possibility to become more physically active, thereby making this the most essential functionality. Those that did not want to use the technology indicated that they believed they were not in the designated target group. They thought of themselves as healthy or stated that they would find it difficult to blend in the use of the technology in their working routines.

Most participants thought that the employer should provide such a technology because it concerns the older employee’s health during working hours. Others thought it was the older employee’s responsibility to look after his /her health and whether to use such a technological aid or not.

Well, I think it would be a good thing when employers offer this service during an annual evaluation. When problems arise. That they can offer this as a possible solution.

Male participant

## Discussion

### Principal Findings

In this article, we have introduced an agile method for testing the end-user acceptance of an eHealth innovation, while in the early stages of development: the EEW. The method has the goal to collect information about end-user acceptance of a new eHealth technology and its main features. This information can help the design team in deciding which features to implement or not, and how to design these functionalities.

We ended the introduction of this article with the following research question: how well does the EEW identify issues that hinder or facilitate end-user acceptance of a future eHealth technology? During the application of the method in two case studies, we learned that it allows participants to understand the workings and use of a future technology, to formulate an opinion about their personal use of a new eHealth technology and to explain their intention to use it. For example, we found that a sleep diary that works with a sensor in the bed led to concerns about privacy. The participants however also provided us with input for devising a control panel for data sharing that allows them to determine themselves who is (not) allowed to inspect this data, thereby making this functionality less privacy-infringing. Next, the evaluations provided the design teams with input for new functionality that would make the technology more valuable to end-users. Finally, the sessions provided input for implementation plans and business models. Participants’ input allowed us to narrow down the designated end-user population and to select the optimal introduction strategy (eg, in our evaluation of the platform for older office workers, the majority opinion was that the employer should provide it, and not purchased by the older office worker him/herself). These experiences allow us to answer the research question positively. Applying the method maps the different factors that hinder or facilitate end-user acceptance of a future eHealth technology and provides (re)design input for improving the technology and the service and business model that accompany it. As such, the EEW is a contribution to the methodological toolkit that design teams can use in the design phase of the different eHealth development frameworks, such as the Center for eHealth Research (CeHReS) roadmap [34] or Integrate, Design, Assess, and Share (IDEAS) [35]. It allows the design team to test whether their implementation of crucial functional requirements is in line with end-user wishes or whether a feature that can be considered to be a technology push is acceptable [36]. By challenging design decisions this way, a technology push without the appropriate amount of user involvement, which the eHealth sector is prone to and which leads to low uptake of the technology [37,38], can be prevented. Ideally, an EEW is conducted whenever there is only a simple prototype of the new technology ready. At this stage, design decisions can be altered at relatively low costs [39]. A side-benefit of involving potential end-users at this early stage is that they will feel committed to the to-be-developed technology, and will, therefore, be more eager to aid the design team throughout the rest of the development process [40].

The type of information that one gathers from an EEW makes it stand out from the popular approaches towards studying end-user acceptance of eHealth. These studies mostly use a quantitative approach and can confirm the influence of factors that are hypothesized to affect end-user acceptance. For example, Zhang and colleagues [41] studied the role of self-efficacy and end-users’ belief in mHealth’s capability to avert negative threats to one’s health. This was done within the context of TAM. From this study, they recommend mHealth developers “to simplify the operations of mHealth services to improve users’ sense of self-efficacy”. Such general advice will be very difficult to translate into specific functionality or interface and interaction design. While studies such as these are beneficial for developing plans for eHealth implementation on a policy level, applying the EEW will be a far more valuable approach for design teams that wish to understand the end-user acceptance of their future eHealth technology and wish to translate this into actionable design recommendations. This finding is in line with the critique that has been voiced about the use of quantitative studies, based on TAM or UTAUT, for informing technology design [17,18]. Using the method can also serve a goal besides generating redesign input. Namely, it can provide insight into what makes up general factors, such as ease of use and perceived usefulness, within the context of eHealth. Alternatively, it can uncover factors that affect acceptance that were previously unknown. As such, using the EEW can enrich the current insights we have about end-user acceptance of eHealth technology.

### Limitations

Of course, the EEW also has its limitations. As the new eHealth innovation has to be explained in simple terms, via a scenario and low-fidelity prototype, it is difficult to put complex technology to the test (eg, decision support functionalities that predict the best treatment options for patients and that apply complicated algorithms). Participants will have a hard time understanding how such sophisticated technology works and what type of output they are being confronted with. For testing these functionalities, interacting with a high-fidelity or Wizard-of-Oz prototype will be far more useful; this allows the prospective end-users to experience the technology directly [42]. Next, when using the technology within the eHealth context, one should be aware of some pitfalls. Some eHealth technologies may provide functionality or health advice that is good for the end-user but not necessarily liked by him or her (eg, the advice to be more physically active instead of watching television). In such cases, end-user feedback should not be taken as the most critical driver for redesign. Another topic is the business model behind the eHealth service. Within the care sector, the means to finance a digital service is often a complicated one and difficult to understand by the individual patient. While the EEW can question willingness to pay from the patient’s perspective, studying financing of the service from multiple perspectives will be difficult. Finally, the generalizability of the results of an EEW is limited. Such evaluations will be done with a limited number of participants and will focus solemnly on one specific eHealth technology. Therefore, one should be very cautious about generalizing the results of an evaluation to eHealth technology in general, or a subset thereof. However, we see the EEW as a method that is to be used for generating redesign input, rather than basic scientific knowledge and thus, do not consider this a significant drawback.

### Conclusions

By introducing the EEW, we have expanded the toolkit of user-centered design methods for eHealth development. The method facilitates (1) easy communication with novices about a future eHealth technology, (2) the identification of factors that can hinder or support end-user acceptance of a future eHealth technology, and (3) an early and cheap possibility for testing functional design decisions.

Previously, acceptance studies were mainly of a confirmative nature, using quantitative methods, which limited their results concerning actionable (re)design advice. Ultimately, the EEW can help to improve unacceptable technology or features that work detrimental for end-user acceptance, and can thereby prevent a mismatch between the needs and expectations of end-users on the one hand, and technological functions on the other. A mismatch that is generally considered to be a significant threat towards the success of eHealth [43,44]. We hope that this article has inspired other researchers to use the EEW as well, and we look forward to learning from their experiences with the method.

## Abbreviations

AAL: European Active and Assisted Living program
CeHReS: Center for eHealth Research
EEW: eHealth End-User Walkthrough
eHealth: electronic health
ICT: Information Communication Technology
IDEAS: integrate, design, assess, and share
TAM: Technology Acceptance Model
UTAUT: Unified Theory of Acceptance and Use of Technology
